# Transcatheter Aortic Valve Implantation (TAVI) Planning with Dual-Layer Spectral CT Using Virtual Monoenergetic Image (VMI) Reconstructions and 20 mL of Contrast Media

**DOI:** 10.3390/jcm13020524

**Published:** 2024-01-17

**Authors:** Federico Fontana, Filippo Piacentino, Aroa Gnesutta, Edoardo Macchi, Andrea Coppola, Angiola Saccomanno, Tonia Gatta, Chiara Recaldini, Manuela Minenna, Claudio Tamborini, Filippo Dossi, Velio Ascenti, Simone Barbera, Giuseppe Cicero, Giulio Carcano, Giorgio Ascenti, Battistina Castiglioni, Massimo Venturini

**Affiliations:** 1 Diagnostic and Interventional Radiology Unit, Circolo Hospital, ASST Sette Laghi, 21100 Varese, Italy; federico.fontana@uninsubria.it (F.F.); agnesutta@studenti.uninsubria.it (A.G.); edoardo.macchi@asst-settelaghi.it (E.M.); asaccomanno@studenti.uninsubria.it (A.S.); tgatta@studenti.uninsubria.it (T.G.); chiara.recaldini@asst-settelaghi.it (C.R.); massimo.venturini@uninsubria.it (M.V.); 2 Postgraduate School of Radiology Technician, Insubria University, 21100 Varese, Italy; manuela.minenna@asst-settelaghi.it; 3 Department of Cardiovascular Diseases, ASST Settelaghi, 21100 Varese, Italy; claudio.tamborini@asst-settelaghi.it (C.T.); filippo.dossi@asst-settelaghi.it (F.D.); battistina.castiglioni@asst-settelaghi.it (B.C.); 4 Postgraduate School of Radiodiagnostics, Policlinico Universitario, University of Milan, 20133 Milano, Italy; velio.ascenti@unimi.it; 5 Diagnostic and Interventional Radiology Unit, Biomorf Department, University Hospital Messina, 98124 Messina, Italy; simone.barbera@studenti.unime.it (S.B.); gcicero@unime.it (G.C.); gascenti@unime.it (G.A.); 6 Department of Medicine and Technological Innovation, Insubria University, 21100 Varese, Italy; giulio.carcano@uninsubria.it

**Keywords:** transcatheter aortic valve implantation, TAVI planning, dual-layer spectral CT, virtual monoenergetic images, iodine load reduction, image quality, patient safety, vascular anatomy assessment

## Abstract

Transcatheter aortic valve implantation (TAVI) is a less invasive alternative to surgical implantation and its implementation is progressively increasing worldwide. We routinely perform pre-procedural aortic angiography CT to assess aortic dimensions and vascular anatomy. This study aims to evaluate the image quality of CTA for TAVI planning using dual-layer spectral CT, with virtual monoenergetic image reconstructions at 40 keV. Thirty-one patients underwent a CTA protocol with the injection of 20 mL of contrast media. Image quality was assessed by measuring the mean density in Hounsfield Units (HU), the signal-to-noise ratio, and the contrast-to-noise ratio in VMI reconstructions. Additionally, a blinded subjective analysis was conducted by two observers. The results showed significant enhancement at all sampled vascular levels with a gradual decrease in HU from proximal to distal regions. Favourable subjective ratings were given for all parameters, with greater variability in the evaluation of iliac axes. A significant negative correlation (*p* < 0.05) was observed between BMI and CA at all vascular levels, indicating reduced contrast enhancement with increasing BMI. Spectral CT, along with reducing iodine load, allows for obtaining high-quality images without a significant increase in noise. The reduction in iodine load can have positive implications in clinical practice, improving patient safety and resource efficiency.

## 1. Introduction

Causes of aortic valve diseases encompass factors such as aging, congenital heart anomalies such as bicuspid aortic valve, conditions affecting the structure of the aorta or heart muscle such as Marfan syndrome, aortic or valvular traumas, and arteritis [1].

Aortic stenosis is the most common valve-related condition, and severe aortic stenosis is prevalent among elderly individuals. While surgical aortic valve replacement is considered the standard of care, TAVI presents an alternative for this high-risk population, as approximately one-third of patients either have contraindications for surgery or opt not to undergo it [2]. 

TAVI is becoming increasingly prevalent due to the advantages of minimally invasive treatments. Prior to the procedure, aortic angiography CT (CTA) with three-dimensional reconstruction is routinely conducted to assess aortic diameters, particularly at the valve level, and to evaluate the anatomical vascular structure. 

This information is crucial for selecting the appropriate size of the surgical device and planning the type of intervention and vascular access. While various approaches can be employed for TAVI, the transfemoral approach is the most used.

Accurate delineation of three-dimensional vascular structures is essential, and enhancing contrast in the aortic region is necessary to achieve high-quality 3D-CTA reconstructions [3]. 

However, the use of iodinated contrast agents in CT carries risks of hypersensitivity allergic reactions, thyroid dysfunction, and nephropathy. To minimize the risk of contrast-induced nephropathy, it is recommended to use the lowest possible dose of contrast agent for diagnostic imaging. 

Conventional single-energy computed tomography (SECT) utilizes a single spectrum of X-ray energy to generate diagnostic images and frequently necessitates repeated scans that expose patients to high levels of radiation [4].

Unlike SECT, dual-energy computed tomography (DECT) relies on capturing two photon spectra, distinguished by high and low energy levels. Various DECT platforms exist, employing different techniques to generate or detect these photon spectra at a high and low energy [5]. DECT utilizes two photon beams with different energy spectra to distinguish materials with similar density but different chemical compositions. The Hounsfield number is correlated with the linear attenuation coefficient (µ), which is not unique for each material but depends on the chemical composition, photon energies, and mass density of the object. The various systems include dual source, twin-beam dual energy, dual layer detector, fast kV switching, and photon counting detector.

In this study, we utilized dual-layer spectral CT, where the acquisition of the two photon spectra occurs simultaneously without introducing additional constraints, except for specific tube voltage values, compared to a conventional helical acquisition [6].

In this context, with dual-layer spectral CT, it is unnecessary to predefine whether spectral reconstructions are desired before the acquisition process [7].

To capture the two photon spectra, the acquisition must be conducted at high tube voltages (120 or 140 kVp). Additionally, as the separation of the energy spectrum takes place at the detector level, an increase in low-energy photon flux is necessary to enhance spectral separation. Both of these adjustments lead to alterations in the energy spectrum of DECT compared to SECT and may impact the radiation dose received by a patient [8]. 

The utilization of two distinct energy levels facilitates improved differentiation of tissues with similar densities. This enhancement is achieved by capitalizing on the photoelectric effect, a phenomenon strongly influenced by both the energy of X-rays and the atomic number of the elements present. 

Post-processing of raw projection data acquired can generate monochromatic virtual images (VMI) with energies ranging from 40 to 140 keV. This is a significant improvement over traditional SECT, which usually operates in the 80 kVp to 140 kVp range and produces images with average energies between 60 keV and 85 keV [9]. Using lower tube voltages (keV) typically results in better contrast in the generated images [10]. This implies that differences in tissue density and composition become more pronounced, facilitating the distinction between various structures within the body. Specifically, this enhances the visibility of iodine-based contrast agents. Conversely, higher tube voltages can help reduce the beam hardening artifact [11]. 

The purpose of this study was to assess the feasibility of an aortic angiography CT with three-dimensional reconstruction for TAVI planning, using a reduced amount of contrast agent and considering both objective and subjective criteria for image evaluation.

## 2. Materials and Methods

Between March 2023 and September 2023, a total of 31 patients with severe aortic stenosis based on clinical and echocardiographic assessments and who were deemed ineligible for surgery, were referred for CTA evaluation prior to TAVI. 

All examinations were performed using a dual-layer spectral CT (IQon Elite Spectral CT, Philips, Healthcare, Amsterdam, The Netherlands). The top layer was constructed using a 1 mm Yttrium-based garnet scintillator, while the bottom layer was composed of gadolinium oxysulphide.

Patients were scanned with the following image parameters: 120 kVp, 565 mAs, 0.200 pitch, 64 × 0.625 mm collimation, helical pitch 1.1. 

The same intravenous contrast agent, Omnipaque (Iohexol 350 mg/mL) and the same quantity (20 mL) was administered to all patients via an 18-gauge peripheral venous access catheter placed in the antecubital vein. The following injection protocol was applied using an automated injector: 3.5 mL/s of a 100 mL mixture containing 20% iodinated contrast agent and 80% physiological saline solution, followed by a saline chaser flush (20 mL). 

The scan was triggered using automatic bolus-tracking with 7-s delay and +80 HU threshold at the level of the thoraco-abdominal aorta. The mean scan time was 27.3 ± 0.3 s (mean ± standard deviation). The chest component of TAVI studies was conducted using retrospective ECG synchronization.

**Figure 1:**
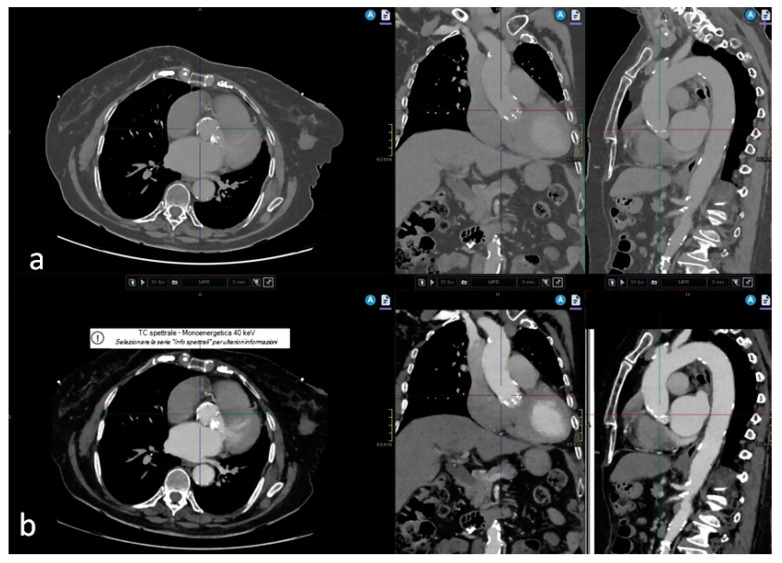
(**a**) Conventional polyenergetic image at 120 kVp, with MPR reconstruction in coronal and sagittal planes; (**b**) respective virtual monoenergetic images (VMI) at 40 keV, also in MPR reconstruction, at the same level.

**Figure 2:**
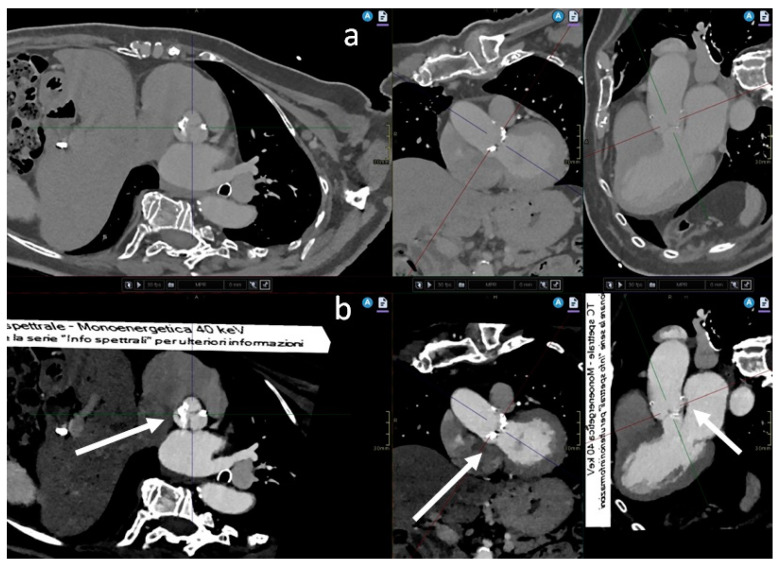
(**a**) Conventional polyenergetic image at 120 kVp, with MPR reconstruction in various planes of space; (**b**) respective virtual monoenergetic images (VMI) at 40 keV, also in MPR reconstruction, at the level of the sinus of Valsalva (white arrow).

Conventional polyenergetic images at 120 kVp were created using iterative reconstruction. The CT images were reconstructed with a 0.9 mm thickness. VMIs were generated from spectral raw data using a specialized workstation (Intellispace Portal, Philips Healthcare, Amsterdam, The Netherlands) at an energy level of 40 keV (Figure 1). These VMIs were also reconstructed with the same 0.9 mm thickness. This energy level was selected because it enabled better contrast visibility without a notable rise in noise levels. The images were reconstructed at 70% of the R-R interval, during diastole for morphologic evaluation of the aortic root and at 40% of the interval, during systole to evaluate measurements of the aortic annulus (Figure 2).

All examinations were accessed from the Picture Archiving and Communication System (PACS); the quantitative and qualitative analyses were evaluated only in VMIs at 40 keV.

**Figure 3:**
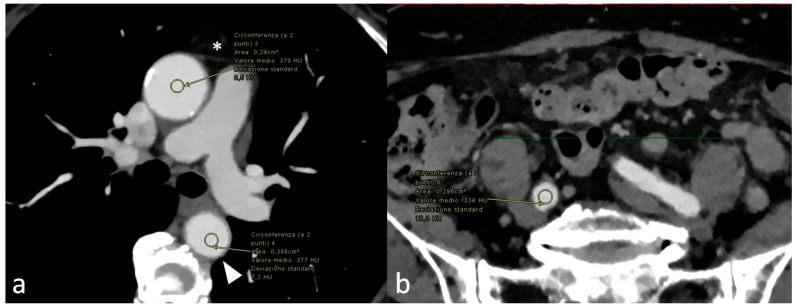
(**a**) Virtual monoenergetic images (VMI) at 40 keV with (region of interest) ROI placement within the ascending thoracic aorta (white asterisk) and the descending thoracic aorta (white arrowhead); (**b**) VMIs at 40 keV with ROI placement within the common iliac artery in the same patient.

For quantitative evaluation, a first radiologist measured contrast attenuation (CA), represented by the mean HU, and noise (standard deviation of CT numbers in HU) with a circular region of interest (ROI) of 3 mm within the left ventricle, ascending aorta, descending aorta, abdominal aorta (at the level of renal arteries), and common iliac artery, and a circular ROI of 2 mm within the external iliac artery (Figure 3). Mean CA and mean noise levels were calculated for all patients. The CA of the psoas muscle was also measured at the same slice of the abdominal aorta for all patients.

Quantitative data were presented as the mean ± standard deviation.

The signal-to-noise ratio (SNR) was calculated as mean CA/mean noise, and the contrast-to-noise ratio (CNR) was calculated as (mean CA of vessel − mean CA of psoas muscle)/mean noise.

Two radiologists, respectively with 18 and 10 years of experience in interpreting vascular imaging, independently reviewed the multiplanar reformation CTA images, reconstructed at 40 keV. They were blinded to the CT scanning parameters. Image quality was evaluated using a subjective five-point scale, considering contrast attenuation, noise, the feasibility of TAVI measurements, and the viability of the iliac route (1—non diagnostic; 2—poor quality; 3—moderate quality; 4—good quality; 5—excellent quality).

Pearson’s correlation coefficient was used to assess the linear correlation between BMI and CA/SNR/CNR, which ranges from −1 (indicating a negative correlation) to 1 (indicating a positive correlation), with 0 indicating no correlation. T-tests were used to evaluate the significance level (*p*-value). A value of *p <* 0.05 was considered statistically significant. All statistical analyses were performed using dedicated software (IBM SPSS Statistics 29.0.1.0). 

## 3. Results

### 3.1. Population Characteristics

Thirty-one patients (mean age: 80.97 ± 6.59; range: 66–94), including 17 males (mean age: 79.06 ± 7.06; range: 66–88 years) and 14 females (mean age: 83.14 ± 5.46; range: 77–94) affected by severe aortic stenosis were enrolled. The mean BMI was 23.38 ± 3.6 (females: 21.63 ± 4.06; males: 25.68 ± 2.30). 

### 3.2. Quantitative and Qualitative Assessment 

**Figure 4:**
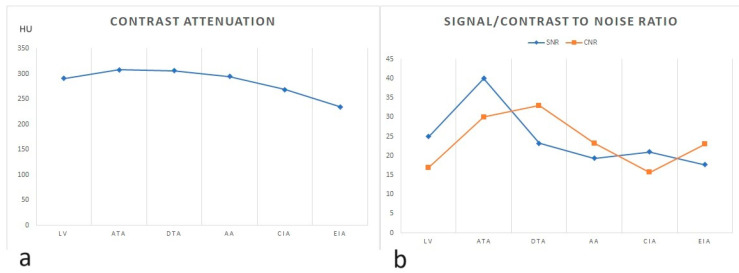
(**a**) Line chart of contrast enhancement progression (y) at various arterial levels (x); (**b**) line chart of signal-to-noise ratio (SNR) (blue line) and contrast-to-noise ratio (CNR) (orange line) progression at various arterial levels.

The mean attenuation of the VMIs at 40 keV images in the left ventricle (LV), ascending thoracic aorta (ATA), descending thoracic aorta (DTA), abdominal aorta (AA), common iliac artery (CIA), and external iliac artery (EIA) were respectively, 290.77 ± 68.65 HU, 308.19 ± 56.53 HU, 305.97 ± 58.54 HU, 294.48 ± 58.49 HU, 268.51 ± 61.22 HU, and 234.22 ± 68 HU (Table 1). This indicates acceptable enhancement at all sampled levels, with a gradual density (HU) decrease from the proximal to distal vascular region (Figure 4). SNR and CNR values at the same levels are listed in the table, which demonstrate a decrease in the signal-to-noise ratio and contrast-to-noise ratio in the more distal vascular levels, with optimal values observed in the segment of the ascending thoracic aorta (Figure 4). 

**Figure 5:**
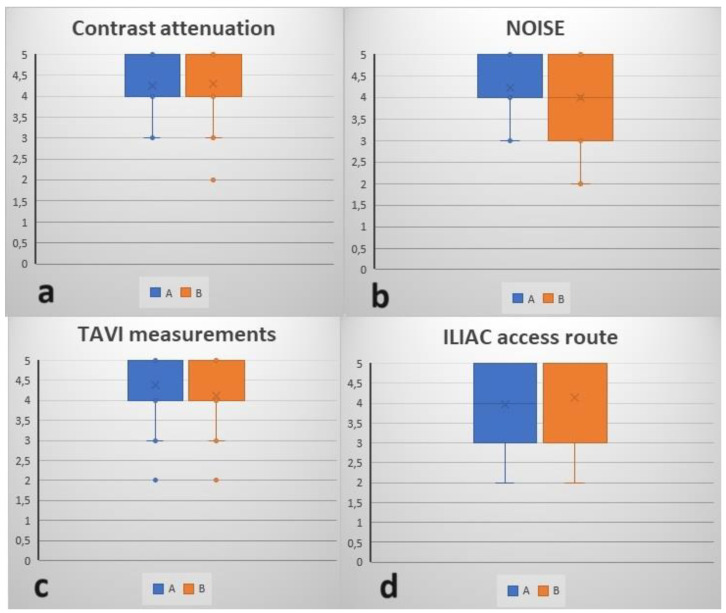
Box plots with subjective scores provided by radiologist A and radiologist B regarding (**a**) contrast attenuation, (**b**) noise, (**c**) feasibility of transcatheter aortic valve implantation (TAVI) measurements, and (**d**) feasibility of iliac route evaluation.

The subjective score for image quality of radiologist A and radiologist B, respectively were 4.26 ± 0.77 and 4.29 ± 0.86 for subjective contrast attenuation, 4.23 ± 0.80 and 4.00 ± 0.97 for subjective image noise, 4.39 ± 0.84 and 4.13 ± 0.96 for the feasibility of TAVI measurements, and 3.97 ± 0.98 and 4.13 ± 1.06 for the feasibility of iliac route evaluation (Table 2). This highlighted a favourable subjective assessment for all parameters, with lower ratings and greater variability in the evaluation of iliac axes. This confirms the results obtained in the objective analysis (Figure 5). 

**Figure 6:**
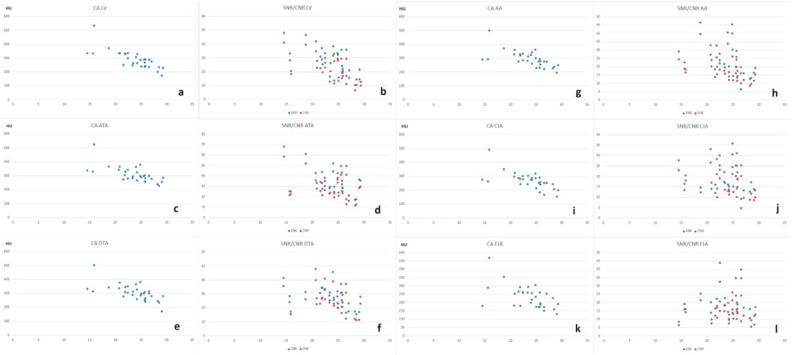
(**a**,**c**,**e**,**g**,**i**,**k**) Scatter plots showing the relationship between contrast attenuation and BMI at various aortic levels; (**b**,**d**,**f**,**h**,**j**,**l**) scatter plots showing the relationships between signal-to-noise ratio (SNR) (blue points) and contrast-to-noise ratio (CNR) (red points) with BMI at various aortic levels.

The trends of CA, SNR, and CNR were examined in relation to the increasing BMI. This revealed a moderate to high negative correlation concerning CA at all analyzed vascular levels, indicating a reduction in contrast enhancement with increasing BMI (*p <* 0.001), with a Pearson correlation coefficient of −0.77 at LV, −0.66 at ATA, −0.65 at DTA, −0.65 at AA, −0.65 at CIA, and –0.56 at EIA. For SNR and CNR, there was a moderate negative correlation at the LV (*p <* 0.001) with Pearson correlation coefficients of −0.58 and −0.62, respectively. At the ATA level, the correlation was moderate for SNR (*p* 0.049) and CNR (*p* 0.031) with Pearson coefficients of −0.36 and −0.39, respectively. At the DTA level, the correlation was moderate for SNR (*p* 0.036) and CNR (*p* 0.019) with Pearson coefficients of −0.38 and −0.42, respectively. For the AA level, the correlation was moderate for SNR (*p* 0.037) and CNR (*p* 0.026) with Pearson coefficients of −0.37 and −0.40, respectively. At the common iliac artery level, the correlation was low with Pearson coefficients of −0.23 for SNR and −0.32 for CNR, and it was absent at the external iliac artery, with Pearson correlations of −0.02 and 0.03 (Figure 6).

## 4. Discussion

Aortic valve stenosis stands as the most prevalent valvular heart disease in the western world, primarily attributed to the aging process and increased life expectancy [12]. The gold standard for treating aortic stenosis involves open-chest surgery with a stopped heart. However, advanced age has presented a substantial impediment to surgical intervention for elderly patients. Medical advancements, such as transcatheter aortic valve implantation, have emerged as a promising treatment option that enhances the clinical course of aortic stenosis without an increased surgical risk [13]. The procedure involves replacing the aortic valve with a bioprosthetic valve inserted via a nonsurgical endovascular (retrograde), transaortic, or transapical (both anterograde) pathway. Risk factors for this procedure include prominent atherosclerotic calcifications, a small native vessel diameter, and marked tortuosity of the iliac arteries [14]. The choice of access route depends on factors such as the device selected, the physical properties of the delivery system, and the suitability of the pathway. The selection of the access route is crucial for the success and eligibility of the procedure. Multidetector CT scans are important for evaluating potential access routes and identifying any issues that could affect the chosen access strategy [15,16]. 

Prior to the procedure, CTA with three-dimensional reconstruction is routinely conducted to assess aortic diameters, particularly at the valve level, and evaluate the anatomical vascular structure. Accurate delineation of three-dimensional vascular structures is crucial, and enhancing contrast in the aortic region is necessary to achieve high-quality 3D-CTA reconstructions. To obtain images with the best quality and minimal motion artifacts, ECG gating is necessary.

Unlike conventional single-energy computed tomography, dual-energy CT utilizes two photon beams with different energy spectra to differentiate materials of similar density but varying chemical compositions. The CT number is determined by the linear attenuation coefficient (µ), which is not unique to each material but depends on chemical composition, photon energies, and the object’s mass density [17]. 

In contrast to other dual-energy imaging methods, dual-layer spectral CT eliminates the need to pre-determine the desire for spectral reconstructions before acquisition. The machine consistently acquires both photon spectra simultaneously, and spectral images can be reconstructed as needed at any time after completing the examination.

From a single study, it is possible to decide retrospectively to enhance contrast by employing VMI reconstructions, create a virtual non-contrast image [18] to minimize patient radiation exposure, or investigate tissue composition through the utilization of Z effective images [19,20]. With dual-layer technology, there is no need to schedule a separate examination for a specific clinical question. In the reconstruction phase, the system can be employed for various inquiries, adjusting to the most suitable modalities to address multiple questions.

As mentioned in the introduction, to acquire the two photon spectra the acquisition must be performed at high tube voltages; furthermore, as the energy spectrum separation takes place at the detector level, it is essential to increase the flux of low-energy photons to enhance spectral differentiation. Van Ommen F. et al. in their study [8] assess the radiation dose linked to dual-energy acquisitions in routine clinical practice across various clinical protocols employing dual-layer spectral CT (DLCT; IQon Spectral CT, Philips Healthcare, the same employed in our study) in comparison to a single-layer detector CT scanner. The results revealed that, for the CTA heart protocol, the mean dose levels on the SLCT were slightly lower than those on the DLCT, whereas for the CTA abdomen, no significant differences were observed between both systems.

In our study, virtual monoenergetic images were used that resulted from reconstructing data at a hypothetical energy level, such as that achieved with a monoenergetic X-ray beam. In recent times, there has been a growing use of low-energy VMIs, especially in situations with limited contrast, such as in oncology and vascular assessment [18,21,22,23]. This involves adjusting the energy level close to the iodine absorption K-edge (around 36 keV) to produce low-energy VMIs. In our study, we opted for images at 40 keV, which provide excellent vascular contrast [24]. However, it is essential to be aware that low-energy VMI reconstructions may introduce more image noise, especially in areas with strong iodine attenuation. Consequently, when selecting the VMI reconstruction energy, various factors should be considered, including the intended purpose of the reconstruction (e.g., improved vascular contrast or mitigation of attenuation-related artefacts), patient size, and optimizing image quality [25]. 

In the literature, we identified four studies that employed dual-energy CT for pre-TAVI assessments. Dubourg et al. [26] compared imaging acquisition using SECT with 60 mL of contrast medium to DECT with 30 mL of contrast. They noted higher CA, SNR, and CNR in the standard protocol, but a lower radiation dose in the DECT protocol at 375 mA. Martin et al. [27] conducted 47 CTA scans with DECT, reconstructing the examinations with VMI at 10 keV intervals (40–100 keV). They identified 40 keV as the energy level with the optimal SNR and CNR. Mangold et al. [28] compared DECT and SECT acquisitions with 80 mL of contrast medium, utilizing VMI reconstructions at 40 keV, and assessed CA, SNR, and CNR. They found high objective image quality and a significant reduction in radiation dose with DECT. Finally, Langenbah et al. [29] studied four patient groups using four different contrast medium doses (60, 50, 40, and 30 mL) and conducted both subjective and objective image evaluations. They determined that 40 keV was the energy level with better results, allowing for a reduction in contrast medium down to 40 mL without compromising diagnostic image quality.

In our study, VMI at 40 keV revealed acceptable enhancement at all sampled vascular levels, with a gradual decrease in HU from proximal to distal vascular levels. Moreover, the SNR and CNR values showed a decreasing trend in the more distal vascular segments, with the highest values found in the ascending thoracic aorta, the region of interest in TAVI planning measurements. The subjective assessment confirmed the objective evaluation with favourable ratings for all parameters. However, it is important to note that the assessment of the iliac route, important in the case of transfemoral access, showed greater variability in scores. This variability may stem from the inherent subjectivity in human interpretations of medical images. This progressive decrease in enhancement and SNR/CNR ratios in the more distant vascular segments might be attributed to a diminished cardiac output in the elderly as opposed to younger patients, with delayed vascular opacification. One of the key aspects of our study was the exploration of the correlation between BMI and imaging parameters (CA, SNR, and CNR). The findings revealed a significant negative correlation between BMI and CA at all vascular levels, suggesting that as the BMI increased, contrast enhancement decreased. This observation holds particular relevance in the realm of personalized medicine, implying that adjusting contrast agent dosages based on BMI may be necessary to uphold optimal image quality. The strongest negative correlation was observed for values below a BMI of 21 and above a BMI of 27, with similar results for intermediate BMIs. This should be considered when deciding on the contrast quantity for overweight patients, as they may not derive substantial benefits from using VMI.

The study is subject to certain limitations, including its single-center design and the relatively small size of the patient sample. Additionally, it was not feasible to conduct a comparative investigation using a standard quantity of contrast agent. This constraint stems from the pilot nature of the study, which aimed to assess the feasibility and quality of the protocol, laying the groundwork for subsequent research with a larger patient cohort and a comparative evaluation.

Preventing contrast-induced nephropathy remains a significant challenge in TAVI procedures. This challenge arises because TAVI typically involves elderly patients with frequently impaired renal function [30,31]. Additionally, TAVI-related procedures often require various examinations that involve the use of intravenous and intra-arterial injections of iodinated contrast agents [32]. It has been suggested that intra-arterial contrast medium administration during catheter-based angiography is associated with a higher incidence of contrast-induced acute kidney failure [33,34]. Reducing the amount of contrast medium also reduces the risk of hypersensitivity allergic reactions [35], minimizes potential impacts on thyroid functions in vulnerable populations such as patients with impaired renal function [36], and enables cost reduction. 

## 5. Conclusions

In conclusion, this study offers valuable insights into essential considerations in the context of contrast-enhanced imaging, especially in patients undergoing TAVI. The findings suggest that reducing the iodine load using lower contrast volumes and employing dual- layer spectral CT at lower energy levels show potential for enhancing the safety and effectiveness of imaging procedures.

## Figures and Tables

**Table 1:** Patients’ contrast attenuation, signal-to-noise ratio (SNR), and contrast-to-noise ratio (CNR) at left ventricle (LV), ascending thoracic aorta (ATA), descending thoracic aorta (DTA), abdominal aorta (AA), common iliac artery (CIA), and external iliac artery (EIA); and patients’ body mass index (BMI). All data except BMI are means ± standard deviation.

	PSOAS	LV	SNR/CNR	ATA	SNR/CNR	DTA	SNR/CNR	AA	SNR/CNR	CIA	SNR/CNR	EIA	SNR/CNR	BMI
1	68 (12)	300 (10)	30/23.2	280 (8)	35/26.5	304 (9)	33.7/26.2	287 (12)	23.9/18.2	279 (11)	25.3/19.1	263 (6)	43.8/32.5	22.4
2	52 (19)	248 (15)	16.5/13	283 (10)	28.3/23.1	290 (10)	29/23.8	282 (13)	21.7/17.7	244 (11)	22.1/17.4	261 (14)	18.6/14.9	23.3
3	72 (10)	333 (9)	37/29	306 (7)	43.7/33.4	310 (9)	34.4/26.4	328 (10)	32.8/25.6	282 (10)	28.2/21	292 (13)	22.4/16.9	21.9
4	57 (16)	333 (12)	27.75/23	366 (11)	33.2/28	376 (14)	26.8/22.7	361 (15)	24/20.2	323 (19)	17/14	253 (26)	9.7/7.5	20.9
5	70 (9)	335 (10)	33.5/26.5	343 (9)	38.1/30.3	350 (8.5)	41.1/33	334 (12)	27.8/22	301 (10)	30.1/23.1	258 (12.5)	20.6/15	22.4
6	56 (18)	199 (22)	9/6.5	252 (16)	15.7/12.2	287 (16)	17.9/14.4	264 (19)	13.9/10.9	281 (22)	12.7/10.2	222 (17)	13/9.7	25.6
7	75 (18)	200 (19)	10.5/6.6	240 (14)	17/11.7	245 (14)	17.5/12.1	228 (18)	12.6/8.5	243 (19)	12.7/8.8	226 (14)	16.1/10.7	26.0
8	82 (20)	238 (14)	17/11.1	300 (17)	17.6/12.8	314 (19)	16.5/12.2	223 (22)	10.1/6.4	167 (18)	9.2/4.7	151 (17)	8.8/40	26.6
9	65 (18)	234 (16)	14.6/10.5	229 (13)	17.6/12.6	233 (15)	15.5/11.2	240 (18)	13.3/9.7	206 (12)	17.1/11.7	160 (17)	9.4/5.5	28.5
10	50 (24)	290 (14)	20.7/17.1	283 (10)	28.3/23.3	264 (9)	29.3/23.7	280 (16)	17.5/14.3	259 (14)	18.5/14.9	190 (10)	19/14	21.6
11	70 (15)	251 (8)	31/22.6	274 (8)	34.2/25.5	278 (9)	30.8/23.1	278 (15)	18.5/13.8	289 (18)	16/12.1	266 (16)	16.6/12.2	29.3
12	50 (17)	230 (18)	12.7/10	287 (8)	35.8/29.6	281 (10)	28.1/23.1	250 (13)	19.2/15.3	200 (15)	13.3/10	191 (11)	17.3/12.8	23.4
13	58 (10)	263 (10)	26.3/20.5	297 (10)	29.7/23.9	311 (10)	31.1/25.3	313 (16)	19.5/15.9	304 (18)	16.8/13.6	295 (13)	22.6/18.2	27.1
14	69 (18)	275 (13)	21.1/15.8	279 (13)	21.4/16.1	280 (12)	23.3/17.5	275 (17)	16.1/12.1	252 (19)	13.2/9.6	253 (15)	16.8/12.2	24.7
15	72 (17)	329 (13)	25.3/19.7	330 (12)	27.5/21.5	346 (10)	34.6/27.4	320 (16)	20/15.5	242 (17)	14.2/10	180 (10)	18/10.8	25.7
16	54 (10)	268 (11)	24.3/19.4	303 (7)	43.2/35.5	309 (10)	30.9/25.5	296 (10)	29.6/24.2	280 (9)	31.1/25.1	261 (13)	20/15.9	20.7
17	38 (20)	240 (15)	16/13.4	289 (12)	24/20.9	299 (13)	23/20	229 (16)	14.3/11.9	214 (14)	15.2/12.5	172 (13)	13.2/10.3	20.7
18	57 (5)	336 (8)	42/34.8	343 (8)	42.8/35.7	337 (7)	48.1/40	329 (10)	32.9/27.2	297 (9)	33/26.6	182 (9)	20.2/13.8	14.5
19	47 (7)	336 (7)	48/41.2	340 (5)	68/58.6	333 (8)	41.6/35.7	291 (10)	29.1/24.4	277 (10)	27.7/23	180 (21)	8.5/6.3	15.6
20	49 (10)	333 (10)	33.3/28.4	331 (13)	25.4/21.7	314 (11)	28.5/24.1	294 (13)	22.6/18.8	263 (16)	16.4/13.4	288 (15)	19.2/15.9	22.0
21	44 (10)	265 (8)	33.1/27.6	267 (9)	29.6/24.7	258 (10)	25.8/21.4	261 (13)	20.1/16.7	274 (18)	15.2/12.8	218 (17)	12.8/10.2	24.2
22	54 (9)	307 (8)	38.4/31.6	364 (7)	52/44.3	366 (8)	45.7/39	325 (8)	40.6/33.9	274 (17)	16.1/13	198 (10)	19.8/14.4	26.6
23	52 (6)	288 (8)	36/29.5	298 (6)	49.6/41	299 (8)	37.3/30.9	277 (14)	19.8/16.1	253 (10)	25.3/20.1	173 (5)	34.6/24.2	25.7
24	53 (9)	238 (12)	19.8/15.4	258 (8)	32.2/25.6	250 (12)	20.8/16.4	233 (9)	25.9/20	184 (13)	14.1/10.1	175 (12)	14.6/10.2	25.8
25	33 (10)	244 (11)	22.2/19.2	259 (9)	28.8/25.1	240 (10)	24/20.7	254 (19)	13.4/11.6	241 (11)	21.9/18.9	196 (12)	16.3/13.6	25
26	43 (13)	368 (11)	33.4/29.5	380 (10)	38/33.7	382 (12)	31.8/28.2	363 (8)	45.4/40	319 (13)	24.5/21.2	305 (19)	16/13.8	24.9
27	42 (6)	323 (10)	32.3/28.1	299 (7)	42.7/36.7	284 (12)	23.6/20.2	303 (10)	30.3/26.1	286 (8)	35.7/30.5	234 (9)	26/21.3	24.4
28	51 (18)	330 (24)	13.7/11.6	326 (14)	23.3/19.6	328 (12)	27.3/23.1	342 (24)	14.2/12.1	288 (21)	13.7/11.3	257 (27)	9.5/7.6	24.2
29	55 (11)	372 (8)	46.5/39.6	366 (6)	61/51.8	344 (11)	31.3/26.3	372 (8)	46.5/39.6	350 (24)	14.6/12.3	354 (14)	25.3/21.3	18.7
30	57 (7)	173 (8)	21.6/14.5	256 (7)	36.6/28.4	170 (10)	17/11.3	197 (12)	16.4/11.6	152 (11)	13.8/8.6	132 (11)	12/6.8	26.4
31	55 (28)	535 (26)	20.6/18.5	526 (21)	25/22.4	503 (29)	17.3/15.5	500 (27)	18.5/16.5	490 (24)	20.4/18.1	469 (29)	16.2/14.3	15.9
Mean		290.7	24.9/16.9	308.2	40/30	305.9	23.2/3	294.5	19.3/23.2	268.5	21/15.6	234.2	17.7/23	23.4
SD		68.6	10.7/6.8	56.5	18.5/10.1	58.5	4.9/9.2	58.5	5/12.1	61.2	10.4/5.5	68	4.7/14.7	3.6

**Table 2:** Subjective scores for radiologist A and B regarding contrast attenuation (CA), noise, feasibility of transcatheter aortic valve implantation (TAVI) measurements, and feasibility of iliac route evaluation.

	CA		NOISE		TAVI Measurements		ILIAC Route	
	A	B	A	B	A	B	A	B
1	3	3	3	4	3	2	4	4
2	4	4	5	5	4	2	5	5
3	3	3	4	3	5	3	3	2
4	4	4	3	2	4	4	4	4
5	5	5	4	3	5	5	4	5
6	4	4	5	3	5	4	3	4
7	5	5	5	5	5	5	4	5
8	4	5	3	3	5	3	4	3
9	3	4	4	3	5	4	3	5
10	3	3	5	5	3	3	2	2
11	3	2	5	5	5	4	3	3
12	4	3	3	2	4	4	4	3
13	4	5	5	5	5	5	5	5
14	5	5	5	5	4	4	4	5
15	5	5	3	4	3	4	5	5
16	4	4	5	5	4	5	5	5
17	5	4	4	4	3	5	5	4
18	5	5	4	4	2	2	3	3
19	4	5	5	5	5	4	2	2
20	5	5	3	3	4	4	5	5
21	5	5	4	4	5	4	4	4
22	5	5	5	5	5	5	5	5
23	4	4	5	4	4	5	2	3
24	4	5	4	4	5	5	4	5
25	5	4	4	3	5	4	5	5
26	5	5	5	5	5	5	5	5
27	4	4	4	4	5	5	4	5
28	5	5	4	4	5	5	4	4
29	5	5	5	5	5	5	5	5
30	3	3	5	5	5	5	3	3
31	5	5	3	3	4	4	5	5
Mean	4.26	4.29	4.23	4	4.39	4.13	3.97	4.13
SD	0.77	0.86	0.80	0.97	0.84	0.96	0.98	1.06

## Data Availability

Data is contained within the article.
